# DNA damage and senescence in osteoprogenitors expressing Osx1 may cause their decrease with age

**DOI:** 10.1111/acel.12597

**Published:** 2017-04-12

**Authors:** Ha‐Neui Kim, Jianhui Chang, Lijian Shao, Li Han, Srividhya Iyer, Stavros C. Manolagas, Charles A. O'Brien, Robert L. Jilka, Daohong Zhou, Maria Almeida

**Affiliations:** ^1^ Division of Endocrinology and Metabolism Center for Osteoporosis and Metabolic Bone Diseases University of Arkansas for Medical Sciences Little Rock AR USA; ^2^ Central Arkansas Veterans Healthcare System Little Rock AR USA; ^3^ Department of Pharmaceutical Sciences University of Arkansas for Medical Sciences Little Rock AR USA

**Keywords:** ABT263, GATA4, NF‐κB, osteoblasts, osteoporosis, p21, p53

## Abstract

Age‐related bone loss in mice results from a decrease in bone formation and an increase in cortical bone resorption. The former is accounted by a decrease in the number of postmitotic osteoblasts which synthesize the bone matrix and is thought to be the consequence of age‐dependent changes in mesenchymal osteoblast progenitors. However, there are no specific markers for these progenitors, and conclusions rely on results from *in vitro* cultures of mixed cell populations. Moreover, the culprits of such changes remain unknown. Here, we have used Osx1‐Cre;TdRFP mice in which osteoprogenitors express the TdRFP fluorescent protein. We report that the number of TdRFP‐Osx1 cells, freshly isolated from the bone marrow, declines by more than 50% between 6 and 24 months of age in both female and male mice. Moreover, TdRFP‐Osx1 cells from old mice exhibited markers of DNA damage and senescence, such as γH2AX foci, G1 cell cycle arrest, phosphorylation of p53, increased p21^CIP^ ^1^ levels, as well as increased levels of GATA4 and activation of NF‐κB – two major stimulators of the senescence‐associated secretory phenotype (SASP). Bone marrow stromal cells from old mice also exhibited elevated expression of SASP genes, including several pro‐osteoclastogenic cytokines, and increased capacity to support osteoclast formation. These changes were greatly attenuated by the senolytic drug ABT263. Together, these findings suggest that the decline in bone mass with age is the result of intrinsic defects in osteoprogenitor cells, leading to decreased osteoblast numbers and increased support of osteoclast formation.

## Introduction

Old age is, by far, the most important risk factor for the development of osteoporosis. In bone biopsies from elderly men and women, the age‐related loss of both cancellous and cortical bone is associated with decreased mean wall thickness – the histomorphometric hallmark of decreased bone formation (Parfitt *et al*., 1995). Loss of bone mass in aged rodents is associated with a decline in the number of osteoblasts, the cells responsible for the synthesis and mineralization of the bone matrix (Almeida *et al*., 2007). Because osteoblasts are postmitotic cells with a short lifespan (Weinstein *et al*., 1998), they need to be constantly replaced with new ones. Osteoblasts arise from progenitors of mesenchymal origin, which express the transcription factors Runx2 and Osterix1 (Osx1) (Park *et al*., 2012).

The decline in the regenerative capacity of most tissues with old age has led to the idea that aging is due, at least in part, to increased cell senescence causing the loss of functional adult stem/progenitor cells (Rossi *et al*., 2008). Cellular senescence is a process in which cells stop dividing and initiate a gene expression pattern known as the senescence‐associated secretory phenotype (SASP) (Campisi, 2013; Lopez‐Otin *et al*., 2013). Several stimuli associated with aging promote senescence. Because the number of senescent cells increases in multiple tissues with aging, it has been widely assumed that senescence contributes to aging (Lopez‐Otin *et al*., 2013; Van Deursen, 2014). Importantly, ablation of senescent cells using genetically modified mice prolongs lifespan and delays age‐related pathologies in naturally aged mice or progeria models (Baker *et al*., 2011, 2016). We have recently shown that senescent cells induced by normal aging or ionizing radiation (IR) can be eliminated by administration of ABT263, a drug that kills senescent cells selectively; and clearance of senescent cells rejuvenates aged tissue stem and progenitor cells (Chang *et al*., 2016).

DNA damage is a major cause of senescence (Mandal *et al*., 2011). Stimulation of p53 and its target gene p21^CIP1^ by the DNA damage response (DDR) plays a fundamental role in the initiation of senescence by causing cell cycle arrest (Roninson, 2002). An alternative or concurrent barrier to proliferation is the derepression of the INK4a/ARF locus and increase in the cyclin inhibitor p16^Ink4a^ (Robles & Adami, 1998). Another component of the DDR is the accumulation of the zinc finger transcription factor GATA4, which in turn stimulates NF‐κB and the SASP (Kang *et al*., 2015). GATA4 protein levels increase in tissues of mice treated with senescence‐inducing stimuli and in multiple mouse and human tissues during physiological aging. The SASP comprises a multitude of pro‐inflammatory cytokines, chemokines, and proteases, which not only reinforce senescence growth arrest in an autocrine manner, but also cause senescence in surrounding cells in a paracrine manner (Coppe *et al*., 2008; Kuilman *et al*., 2008). These factors also cause low‐grade inflammation.

In both humans and rodents, the reduced osteoblast number in the aging skeleton has been attributed to changes in bone marrow‐derived mesenchymal progenitors, including a decrease in the number of mesenchymal stem cells, defective proliferation/differentiation of progenitor cells, increased apoptosis, or increased senescence (Stenderup *et al*., 2003; Sethe *et al*., 2006). Studies using bone marrow‐derived stromal cells as a surrogate for osteoblast progenitors have suggested that serially passaged cells from aged humans or mice become senescence at earlier passages compared to cells from young individuals (Stenderup *et al*., 2003; Zhou *et al*., 2008; Sui *et al*., 2016). However, serial passaging in and of itself causes replicative senescence (Hayflick & Moorhead, 1961) and bone marrow cell cultures are heterogeneous. Therefore, it remains unclear whether the number of senescent osteoblast progenitors increases with old age. Moreover, the contribution of the decline in osteoblast progenitor number to the decrease in bone formation with age remains unknown because of the lack of methods to specifically identify and isolate mesenchymal progenitors. Therefore, the molecular mechanisms responsible for the decline in osteoblast number have remained elusive. To overcome these limitations, we generated a mouse model in which osteoblast progenitors are labeled with a red fluorescent protein to facilitate their isolation by fluorescence‐activated cell sorting (FACS) and examination of the effects of aging in freshly isolated cells. We present evidence that the decline in bone formation with age can be accounted for by a decrease in the number of osteoprogenitors due to DNA damage‐induced cell senescence.

## Results

### The number of osteoprogenitors marked by Osx1‐Cre declines with age

**Figure 1:**
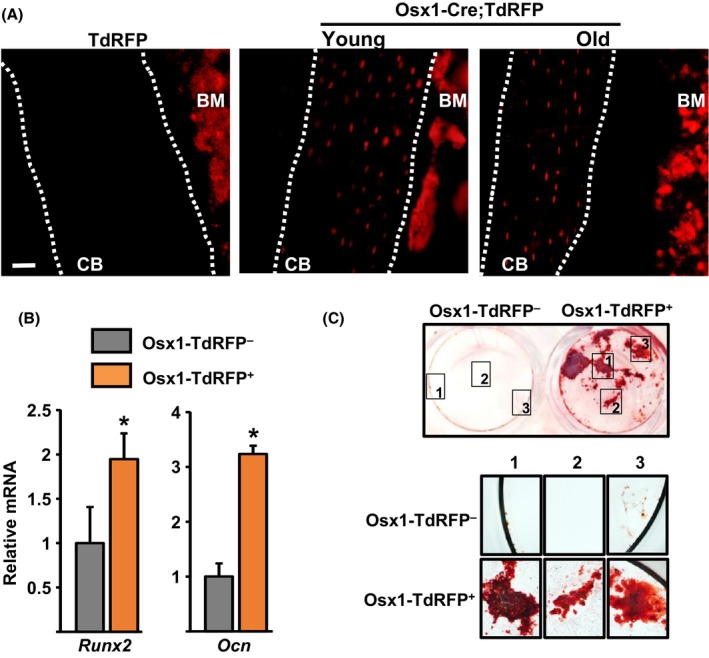
Cells targeted by Osx1‐Cre transgene in the bone marrow. (A) Representative femur sections from 6‐ and 24‐month‐old Osx1‐Cre;TdRFP mice. *CB*, cortical bone; *BM*, bone marrow; scale bar 20 μm. (B and C) Sorted cells from Osx1‐Cre;TdRFP mice (*n* = 6 mice) cultured with ascorbate and β‐glycerophosphate. (B) mRNA levels by qRT‐PCR in cells cultured for 7 days and (C) Alizarin Red S staining in cells cultured for 21 days, (*top*) 2.5× magnification (*bottom*) 20× magnification. *P < 0.05 by Student's *t*‐test. Bars represent mean and SD (*error bars*).

To determine whether osteoblast progenitors change with age, we crossed Osx1‐Cre (Osx1‐GFP::Cre) (Rodda & McMahon, 2006) and stop‐loxP‐tandem dimer red fluorescent protein (Td‐RFP) mice (Luche *et al*., 2007) to generate Osx1‐Cre;TdRFP mice. In these mice, osteoprogenitors and all their descendants, including osteoblasts and osteocytes, express TdRFP. Cells targeted by this Osx1‐Cre transgene are present in the bone marrow of adult mice and represent bipotential progenitors capable of generating osteoblasts and adipocytes (Ono & Kronenberg, 2015). Furthermore, Osx1‐Cre‐expressing cells give rise to all osteoblast and osteocytes in the skeleton of young mice (Xiong *et al*., 2011). Similarly, all osteocytes were TdRFP‐positive in femoral cortical bone of 24‐month‐old Osx1‐Cre;TdRFP mice (Fig. 1A). Cortical bone from TdRFP control mice did not exhibit any red fluorescence; however, some background fluorescence was detected in the bone marrow. Freshly isolated Osx1‐TdRFP^+^ bone marrow cells from Osx1‐Cre;TdRFP mice, using FACS, had elevated levels of Runx2 and osteocalcin compared to Osx1‐TdRFP^–^ cells (Fig. 1B) and formed mineralized nodules when cultured in osteogenic conditions (Fig. 1C).

**Figure 2:**
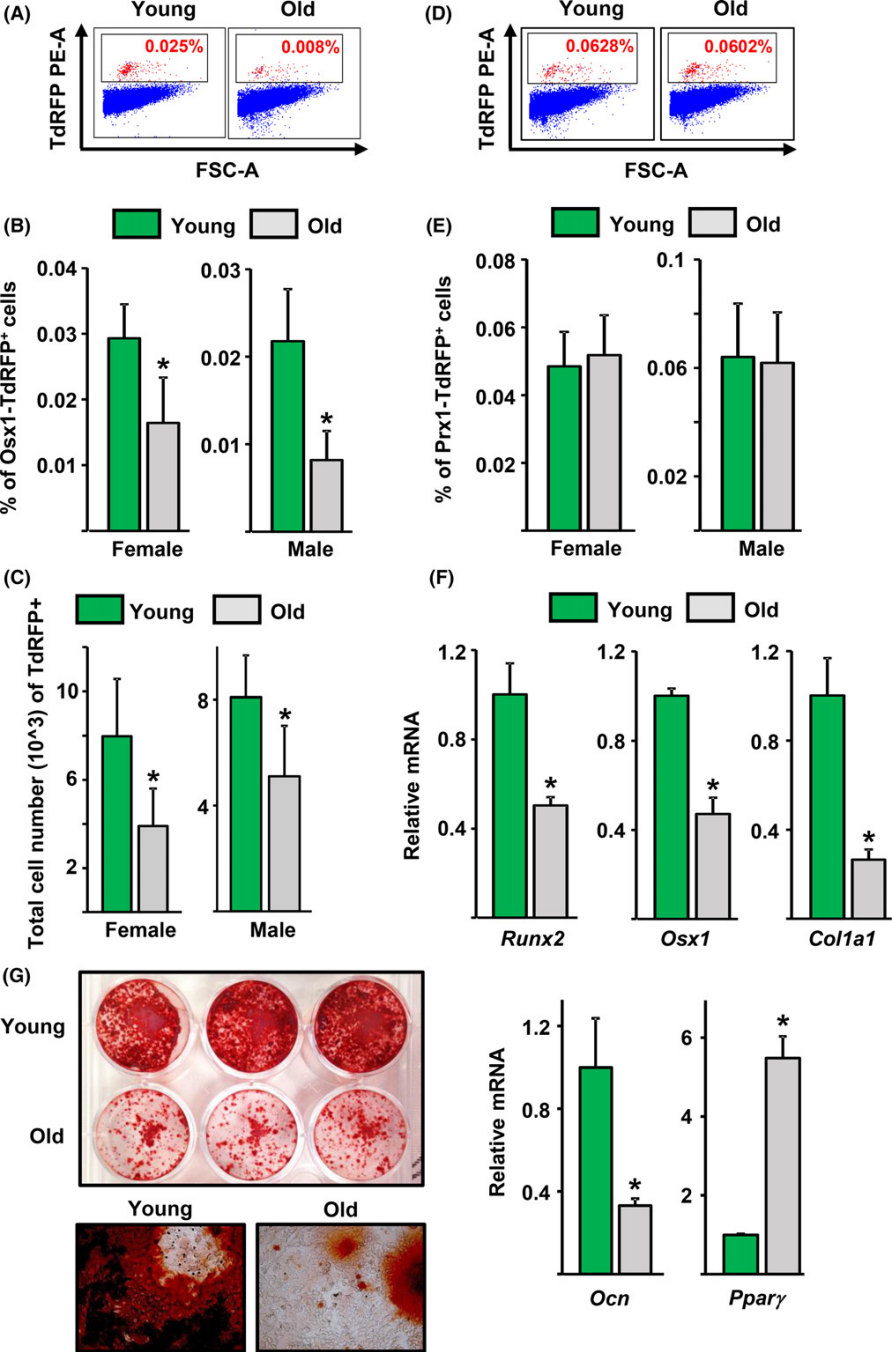
The number of Osx1‐Cre‐expressing cells decline with age. (A‐C) Osx1‐TdRFP ^+^ bone marrow cells were isolated from 4‐ and 23‐month‐old Osx1‐Cre;TdRFP mice (*n* = 4–6 mice/group). (A) Representative flow cytometric analysis of Osx1‐TdRFP ^+^ bone marrow cells. (B) Percentage of Osx1‐TdRFP ^+^ cells in the bone marrow. (C) Number of Osx1‐TdRFP ^+^ cells in the hindlimbs from each mouse. (D and E) Prx1‐TdRFP ^+^ bone marrow cells were isolated from 3‐ and 24‐month‐old Prx1‐TdRFP mice (*n* = 4–5 mice/group). (D) Representative flow cytometric analysis of Prx1‐TdRFP ^+^ bone marrow cells. (E) Percentage of Prx1‐TdRFP ^+^ cells in the bone marrow. (F and G) Bone marrow stromal cells from 6‐ and 26‐month‐old Osx1‐Cre;TdRFP mice cultured with ascorbate and β‐glycerophosphate. (F) mRNA levels by qRT‐PCR in cells cultured for 7 days and (G) Alizarin Red S staining in cells cultured for 21 days (triplicates), (*top*) 2.5× magnification (*bottom*) 20× magnification. *P < 0.05 by Student's *t*‐test. Bars represent mean and SD (*error bars*).

We counted Osx1‐TdRFP^+^ cells in the bone marrow of young adult (4–6 months) mice and found that this population represents about 0.02–0.03% of all cells (Fig. 2A–C). The number of the TdRFP+ cells decreased by approximately 50% in old (20–24 months) female or male mice (average of four independent experiments, one of which is shown in Fig. 2A). We also compared the number of cells marked by a Prx1‐Cre transgene, which targets mesenchymal stem cells at a stage earlier than Osx1, in the bone marrow of young and old Prx1‐Cre;TdRFP mice. Prx1‐TdRFP^+^ cells represented about 0.05–0.06% of all cells in the bone marrow, and their abundance did not change with age (Fig. 2D,E).

Cultured bone marrow stromal cells from old mice exhibited lower mRNA levels of the osteoblast markers Runx2, Osx1, collagen 1a1 (Col1a1), and osteocalcin (Ocn) (Fig. 2F) and formed fewer mineralized nodules than cells from young mice (Fig. 2G), despite the fact that both cultures reached confluence. In contrast, the adipocyte marker Pparγ was increased (Fig. 2F). The decline in osteoblast generation seen in the cultured cells is probably due to the lower number of Osx1‐expressing cells present in the bone marrow of old mice. Because the mineralization assay requires the cells to remain in culture for an extended period of time, it is not possible to attribute the changes in mineralization to a particular cellular event, for example, proliferation, apoptosis, differentiation, or matrix secretion.

### Osteoprogenitors in old mice are arrested at G1 and exhibit increased DNA damage and p53 activation

**Figure 3:**
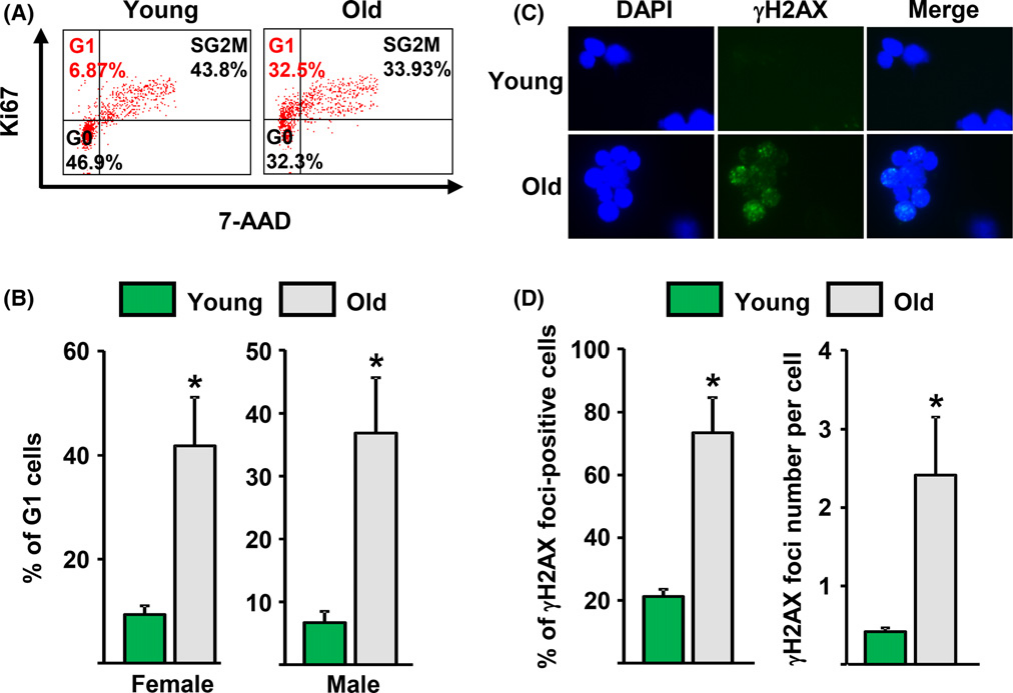
Osx1‐TdRFP ^+^ cells from old mice exhibit DNA damage and G1 arrest. (A) Representative FACS analysis of the cell cycle distribution of Osx1‐TdRFP ^+^ cells from 3‐ to 6‐month‐old and 23‐ to 26‐month‐old Osx1‐Cre;TdRFP mice (*n* = 5 mice/group). (B) Percentage of Osx1‐TdRFP ^+^ cells at G1 in mice described in A. (C and D) Analysis of DNA double‐strand breaks in TdRFP‐Osx1^+^ cells from 6‐ and 25‐month‐old Osx1‐Cre;TdRFP male mice (*n* = 5 mice/group). (C) Representative photomicrographs of γH2AX immunofluorescence staining (green) and nucleic counterstaining with Hoechst‐33342 (blue). (D) Percentage of γH2AX‐positive cells (left) and number of γH2AX foci per cell (right). *P < 0.05 by Student's *t*‐test. Bars represent mean and SD (*error bars*).

We next examined cell cycle distribution of Osx1‐TdRFP^+^ cells. The percentage of cells at the G1 phase in old female and male mice was approximately fivefold higher than in cells from young mice (Fig. 3A,B). Because DNA damage is one of the major causes of cell cycle arrest, we quantified γH2AX foci – a common marker of DNA damage – in the nucleus of Osx1‐TdRFP^+^ cells. To this end, we performed immunostaining in freshly isolated cells from young or old mice. Approximately 70% of the cells isolated from old mice exhibited γ‐H2AX foci as compared to 20% in cells from young mice (Fig. 3C,D). The average number of γH2AX foci per cell also increased with age (Fig. 3D).

**Figure 4:**
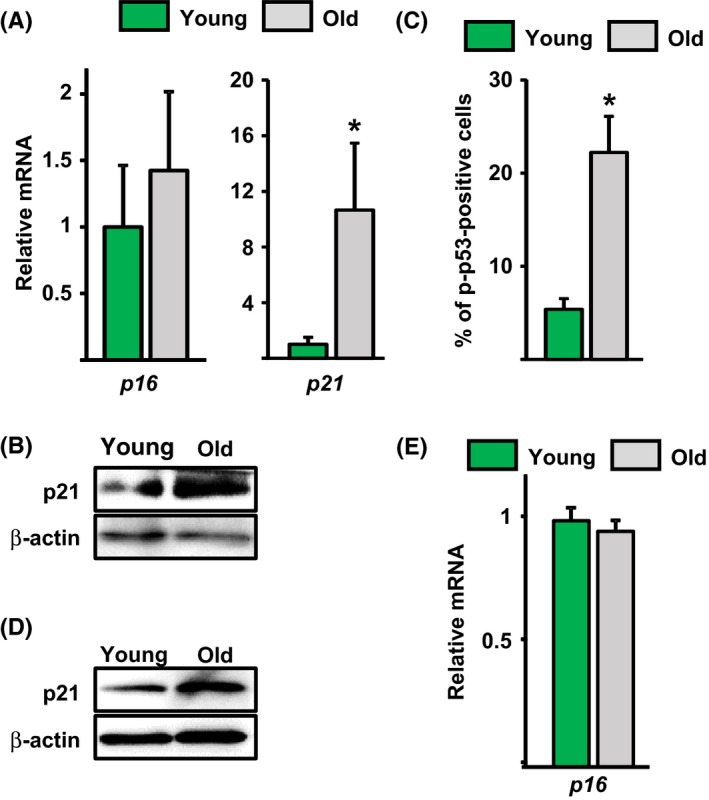
Osx1‐TdRFP ^+^ cells from old mice have increased p53/p21 levels. (A and B) Osx1‐TdRFP ^+^ cells from 6‐ and 24‐month‐old Osx1‐Cre;TdRFP female mice (*n* = 5 mice/group) (A) mRNA levels by qRT‐PCR and (B) protein levels by Western blot. (C) Percentage of p‐p53‐positive cells in Osx1‐TdRFP ^+^ cells from 3‐ and 24‐month‐old Osx1‐Cre;TdRFP male mice (*n* = 5 mice/group). (D and E) Bone marrow stromal cells from 6‐ and 26‐month‐old Osx1‐Cre;TdRFP female mice (*n* = 5 mice/group) cultured with ascorbate and β‐glycerophosphate. (D) Protein levels by Western blot in cells cultured for 10 days and (E) mRNA levels by qRT‐PCR in cells cultured for 7 days. *P < 0.05 by Student's *t*‐test. Bars represent mean and SD (*error bars*).

We next determined whether p16 and/or p21 might mediate the cell cycle arrest seen in osteoprogenitors from old mice. The mRNA levels of p16 were unaffected by age (Fig. 4A). In contrast, both the mRNA and protein levels of p21 were elevated in Osx1‐TdRFP^+^ cells from old mice (Fig. 4A,B). In line with the increase in p21, the number of osteoprogenitors exhibiting p53 phosphorylation increased in old mice, as determined by immunostaining with a Ser15 phospho‐p53 antibody in freshly isolated Osx1‐TdRFP^+^ cells (Fig. 4C). A similar increase in p21 (Fig. 4D), but unaltered p16 levels (Fig. 4E), was seen in cultures of bone marrow cells from old mice. These findings suggest that Osx1‐TdRFP^+^ cell cycle arrest was caused by DNA damage‐induced stimulation of the p53/p21 pathway.

### Osteoprogenitors from old mice exhibit increased GATA4, NF‐κB, expression of the SASP, and increased osteoclastogenic support capacity

**Figure 5:**
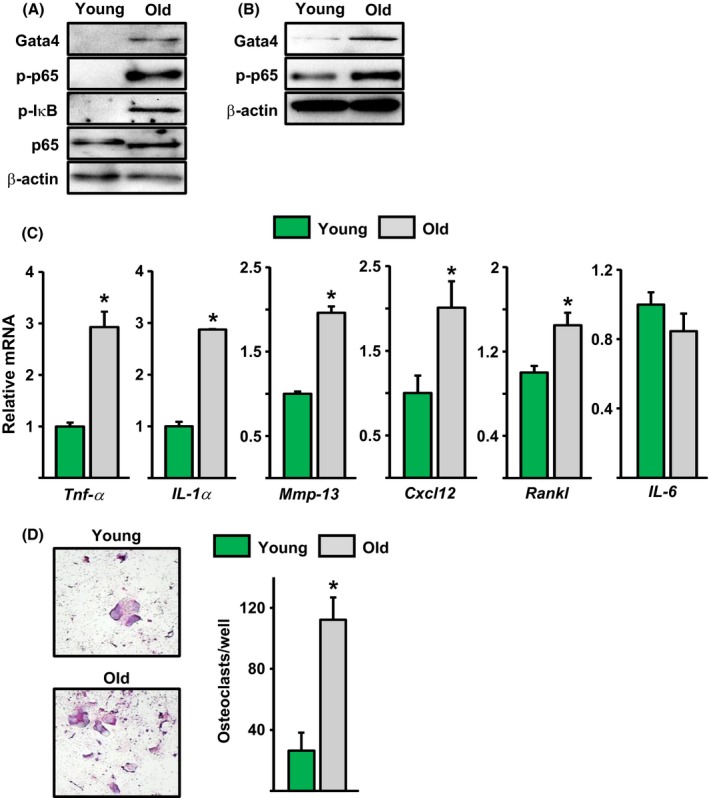
Osteoprogenitors in old mice exhibit elevated GATA4, active NF‐κB, SASP, and osteoclastogenic properties. Protein by Western blot in (A) Osx1‐TdRFP ^+^ cells from 6‐ and 24‐month‐old Osx1‐Cre;TdRFP female mice (*n* = 5 mice/group) and (B) bone marrow stromal cells from 6‐ and 26‐month‐old Osx1‐Cre;TdRFP female mice cultured with ascorbate and β‐glycerophosphate for 7 days (triplicates). (C) mRNA levels by qRT‐PCR in cells described in B. (D) Mouse bone marrow macrophages from 6‐month‐old wild‐type C57BL/6J male mice were co‐cultured with stromal cells from 6‐ and 26‐month‐old wild‐type C57BL/6J male mice in the presence of 1α,25(OH)_2_D_3_ for 7 days, and the cells were fixed and stained for TRAP. TRAP‐positive multinucleated cells containing three or more nuclei were counted as osteoclasts. *P < 0.05 by Student's *t*‐test. Bars represent mean and SD (*error bars*).

We next looked for signs of the SASP. GATA4 protein levels were increased in freshly isolated Osx1‐TdRFP^+^ cells from old mice when compared with cells from young mice (Fig. 5A), as determined by Western blot. Similarly, the levels of p‐IkB and p‐p65 were elevated in cells from old mice, indicating stimulation of NF‐κB signaling. Bone marrow‐derived osteoblastic cells from old mice cultured in osteogenic medium for 7 days also exhibited higher GATA4 and p‐p65 levels than cells from young mice (Fig. 5B). In line with evidence that GATA4/NF‐κB signaling is a major inducer of the SASP, the mRNA abundance of common SASP components, such as TNF‐α, IL‐1α, MMP‐13, CXCL12 but not IL‐6, was elevated in cultured cells from old mice (Fig. 5C). The expression of the critical osteoclastogenic factor RANKL was also elevated. We also examined the ability of stromal cells from old mice to support osteoclast formation. The number of osteoclasts formed in co‐cultures of macrophages with bone marrow‐derived stromal cells, in the presence of 1,25 dihydroxy vitamin D3, was higher when stromal cells originated from old mice compared to cells from young mice (Fig. 5D). Similar findings have been reported by others (Cao *et al*., 2005).

### The senolytic drug ABT263 attenuated the expression of SASP and the osteoclastogenic support capacity of bone marrow stromal cells from old mice

**Figure 6:**
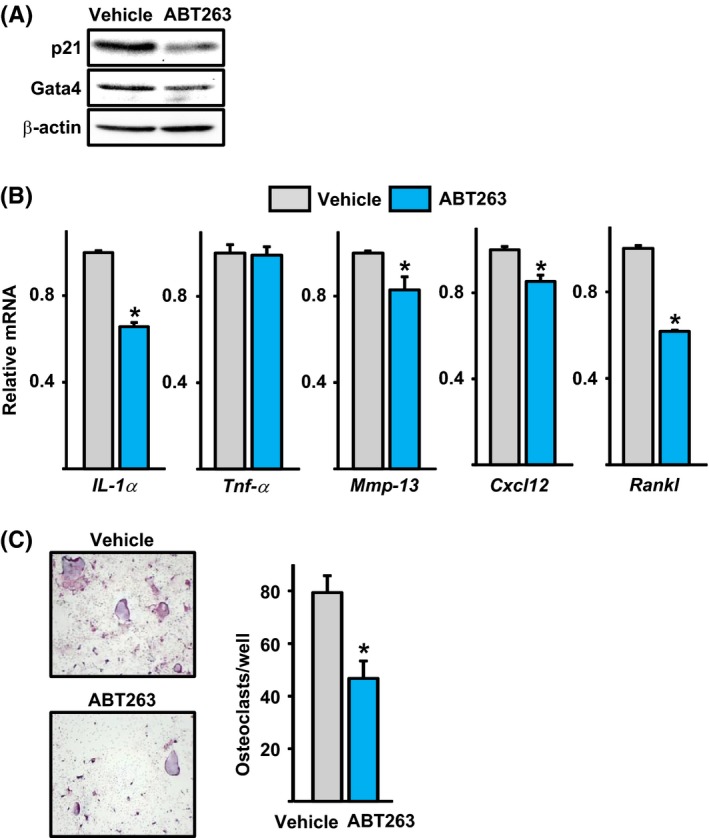
Senescent cell clearance by ABT263 attenuates GATA4 and SASP expression in stromal cell cultures from old mice. (A) Protein by Western blot in bone marrow stromal cells from 24‐month‐old wild‐type C57BL/6J female mice cultured with ascorbate and β‐glycerophosphate for 7 days, following pretreatment with ABT263 (triplicates). (B) mRNA levels by qRT‐PCR in cells described in A. (C) Bone marrow macrophages from 3‐month‐old wild‐type C57BL/6J female mice were co‐cultured for 7 days with stromal cells from 24‐month‐old wild‐type C57BL/6 female mice pretreated with vehicle or ABT263. TRAP‐positive multinucleated cells containing three or more nuclei were counted as osteoclasts. *P < 0.05 by Student's *t*‐test. Bars represent mean and SD (*error bars*).

We have previously shown that ABT263 kills selectively senescent cells (Chang *et al*., 2016). Culture of bone marrow stromal cells from old mice with ABT263 for 5 days, dose dependently, increased apoptosis (data not shown). ABT263 also caused a decrease in protein levels of p21 and GATA4 (Fig. 6A), as well as the expression of IL‐1α, MMP‐13, CXCL12, and RANKL (Fig. 6B). TNF‐α was not affected. Co‐cultures of bone marrow stromal cells from old mice pretreated with ABT263 exhibited lower capacity to support osteoclast formation in co‐cultures with macrophages, compared to cells pretreated with vehicle control (Fig. 6C). These findings suggest that ABT263 can eliminate senescent osteoprogenitor cells.

### Radiation‐induced senescence replicates the effects of aging in cultured osteoblastic cells

**Figure 7:**
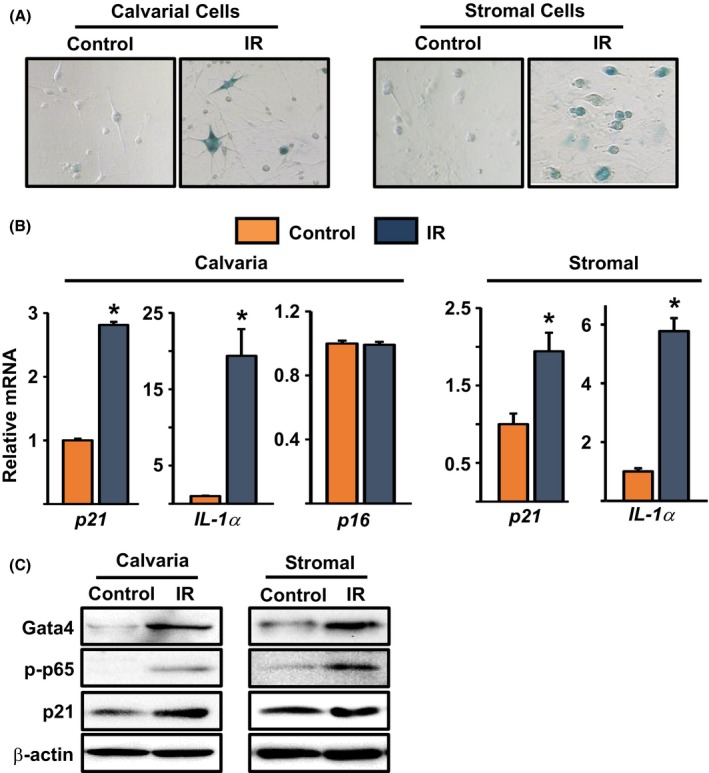
IR induces senescence in osteoprogenitors. (A) SA‐β‐gal staining of IR‐induced senescent cells. (*IR*, 7 days after exposure to 10 Gy). (B) Gene expression in IR‐induced senescent cells. (C) Western blot analysis was performed in IR‐induced senescent cells. *P < 0.05 by Student's *t*‐test. Bars represent mean and SD (*error bars*).

Finally, we determined whether the effects of aging are similar to those seen with other forms of DNA damage. Exposure to ionizing radiation (IR) causes DNA damage and senescence (Shao *et al*., 2014). To determine whether IR could replicate the effects of aging in primary osteoblastic cells, we subjected cultured newborn calvaria cells or bone marrow‐derived stromal cells to 10 Gy of γ‐radiation. After a few passages, the irradiated cells exhibited increases in senescence‐associated β‐galactosidase (SA‐β‐gal) activity (Fig. 7A) and mRNA expression of p21 and IL‐1α (Fig. 7B), while the levels of p16 were unaltered. Western blot analysis confirmed the increased levels of p21 and further revealed that irradiation‐induced senescent calvaria and bone marrow‐derived osteoblasts had a 2.5‐ and twofold increased expression of GATA4, respectively (Fig. 7C). Phospho‐p65 was also increased in both cell types. These findings indicate that induction of DNA damage in cultured osteoblastic cells by IR causes DNA damage responses and senescence similar to the ones seen in osteoprogenitors from old mice.

## Discussion

In the studies described herein, we isolated a homogeneous population of osteoblast progenitors from the bone marrow and determined the effects of old age. We have found that osteoblast progenitors from old mice exhibit several markers of senescence. These senescence features are associated with a decline in the number of osteoblast progenitors and a decrease in matrix synthesizing osteoblast number and bone formation rate, shown previously (Almeida *et al*., 2007). The decline in cell number is specific for the population expressing Osx1. Cells expressing Prx1, which include a broader group of cells such as mesenchymal stem cells, fibroblasts, CXCL12‐abundant reticular (CAR) cells, adipocytes as well as osteoprogenitors, did not decline with age. A decline in Osx1‐expressing cells with age was also noted, but not quantified, by Kusumbe *et al*. (2014) using immunofluorescent staining and confocal microscopy of bone sections. Our findings suggest that the osteoprogenitor cell population marked by Osx1‐TdRFP is susceptible to the damaging effects of aging.

The number of γH2AX foci was increased in osteoprogenitors from old mice, strongly suggesting that DNA damage is elevated in these cells. Several other mammalian tissues show an age‐related increase in foci of γH2AX (Herbig *et al*., 2006; Wang *et al*., 2009). This may result from the decreased tendency of double‐strand breaks to be repaired with aging (White *et al*., 2013), which may contribute to the accumulation of senescent cells, that harbor persistent DNA damage foci (Rodier *et al*., 2011). Previous studies with mouse models of accelerated aging due to defective DNA damage repair have elucidated that accumulation of DNA damage causes several features of early aging including decreased bone formation and low bone mass (Vogel *et al*., 1999; Dolle *et al*., 2011; Saeed *et al*., 2011; Chen *et al*., 2013). DNA damage in these models is caused by a deficiency in telomerase, ERCC1 (Excision Repair Cross Complementary group 1), or Ku80. Similar to physiological aging, the low bone mass in these accelerated aging models is associated with low bone formation. Although freshly isolated osteoprogenitors were not examined, bone marrow stromal cell cultures from ERCC1^−/−^ or Tert^−/−^ mice also exhibit markers of senescence (Saeed *et al*., 2011; Chen *et al*., 2013). Further support for the contention that DNA damage is responsible for osteoprogenitor cell senescence and the decline in bone formation with age are our findings that DNA damage induced by irradiation causes changes in osteoprogenitors that are similar to the ones seen with aging. In addition, DNA damage, due to focal irradiation in long bones, causes senescence in osteoblast lineage cells, decreases bone formation, and leads to bone loss in mice (Chandra *et al*., 2017).

Phosphorylation of p53 and the levels of p21 were greatly increased in osteoprogenitors from old mice, and these changes were associated with a higher number of cells arrested at the G1 phase. An increase in p21 levels due to p53 activation is a critical mediator of cell cycle arrest and senescence following DNA damage in several cell types (Herbig *et al*., 2004; Passos *et al*., 2010). In osteoblastic cells, p21 inhibits proliferation and differentiation (Bellosta *et al*., 2003). Studies using p53^−/−^ mice, or mice with osteoblast specific p53 gain‐ or loss‐of‐function have provided compelling evidence that p53 inhibits osteoblastogenesis (Lengner *et al*., 2006; Wang *et al*., 2006). Furthermore, transgenic mice with global gain‐of‐function mutation of p53 rapidly accumulate senescent cells and exhibit accelerated aging phenotypes, including low bone mass (Tyner *et al*., 2002; Maier *et al*., 2004). Nevertheless, it remains unknown whether gain‐of‐function of p53 causes osteoprogenitor senescence and whether this contributes to the skeletal defects seen in the mice. Interestingly, p16^Ink4a^ levels were unaffected in osteoprogenitors from old mice and in senescent calvaria cells induced by γ‐radiation. These results are in line with evidence that p16 and p21 pathways are triggered independently and that activation of the p53/p21 pathway, but not p16, correlates with the presence of γ‐H2AX foci in response to DNA damage (Herbig & Sedivy, 2006). Furthermore, studies in human cells have shown that some cell types, for example, skin fibroblasts such as BJ cells, do not exhibit p16 up‐regulation with senescence and activation of p53 alone is sufficient to cause the senescence arrest (Itahana *et al*., 2003). Human endothelial cells primarily depend on p21 but not p16 for senescent induction (Wang *et al*., 2016). In contrast, lung‐derived fibroblasts require both p53 and p16 to achieve senescence arrest. Similar to osteoprogenitors, osteocytes of aged mice exhibit markers of senescence and expression of SASP. However, in difference to Osx1‐expressing cells, osteocytes from old mice exhibit elevated p16 (Farr *et al*., 2016). While the reasons for the differences in the expression of p16 and p21 remain unclear, it has been proposed that distinct cell types might utilize different cyclin‐dependent kinase inhibitors to induce senescence, due to activation of different stress response pathways (Herbig & Sedivy, 2006; Wang *et al*., 2016). Together, these findings support the idea that stimulation of the p53/p21 component of the DDR is responsible for cell cycle arrest in osteoprogenitors and contributes to the decline in osteoblast number in the aging skeleton.

GATA4 levels increased with age in osteoprogenitors, as well as with radiation in cultured osteoblastic cells. These observations are in line with evidence that DNA damage promotes GATA4 accumulation (Kang *et al*., 2015). Importantly, stabilization of GATA4 is sufficient to induce senescence and the SASP in cultured human cells. GATA4 increases the SASP, at least in part, via stimulation of NF‐κB activity (Kang *et al*., 2015). Consistent with this evidence, NF‐κB activity was increased in osteoprogenitors; and SASP components such as TNF‐α, MMP13, CXCL12, and IL‐1α were also elevated. All these proteins have osteoclastogenic properties (Pfeilschifter *et al*., 1989; Wright *et al*., 2005; Fu *et al*., 2016) and most probably contributed, along with RANKL, to the increased osteoclast formation seen in co‐cultures with stromal cells from old mice. Support for the contention that senescent cells contribute to osteoclastogenesis is provided by the findings that senescent osteoblastic cells, resulting from overexpression of the cyclin inhibitor p27KIP1, increase osteoclast formation in a co‐culture system *in vitro* and osteoclasts number *in vivo* (Luo *et al*., 2016). It is, therefore, plausible that the SASP from osteoprogenitors and osteocytes contributes to the increased bone resorption that occurs in cortical bone with age (Jilka *et al*., 2014; Ucer *et al*., 2017). In addition, our work suggests that ABT263 can attenuate the SASP and osteoclastogenesis in bone marrow stromal cell cultures from old mice. These results are in line with our previous work showing that ABT263 abrogates the SASP response in bone marrow stromal cells caused by irradiation in mice (Chang *et al*., 2016). Taken together, these findings suggest that ABT263 can eliminate senescent osteoblast progenitors in aged mice. GATA4 stimulation might also contribute to the age‐related decline in bone formation. Indeed, overexpression of GATA4 in primary calvaria cells attenuates Runx2 promoter activity and inhibits osteoblastogenesis (Song *et al*., 2014). In contrast, GATA4 in osteoblasts might contribute to bone mineralization during development (Guemes *et al*., 2014).

Several cell types within the osteoblast lineage have been proposed to implicated in the decline of bone formation with age (Kassem & Marie, 2011; Almeida & O'Brien, 2013). Our present findings indicate that the age‐related decrease in bone formation and increase in bone resorption could be accounted for by intrinsic defects in osteoblast progenitors that lead to a decrease in their number combined with an increase in their capacity to support osteoclast formation because of SASP. Together with evidence that removal of senescent cells via genetic or pharmacologic means attenuates the age‐dependent degeneration of several tissues (Baker *et al*., 2011, 2016; Chang *et al*., 2016), the present work supports the notion that removal of senescent cells may represent a therapeutic approach to the prevention and treatment of involutional osteoporosis.

## Experimental procedures

### Mice

Mice expressing RFP in the pluripotent mesenchymal progenitors or osteoblast progenitors were generated by crossing mice heterozygous with a Prx1‐Cre (Logan *et al*., 2002) or Osx1‐Cre (Rodda & McMahon, 2006) transgene with mice heterozygous or homozygous for an stop‐loxP‐tdTRFP allele (Luche *et al*., 2007) to obtain Prx1‐Cre or Osx1‐Cre;TdRFP mice. Offspring were genotyped by PCR using following primer sequences: Cre‐for, 5′‐GCGGTCTGGCAGTAAAAACTATC‐3′, Cre‐rev, 5′‐GTGAAACAGCATTGC TGTCACTT‐3′, product size 102 bp; tdRFP1 (HL15), 5′‐AAG ACCGCGAAGAGTTTGTCC‐3′, tdRFP2 (HL54) 5′‐TAAGCCTGCCCAGAAGACTCC‐3′, tdRFP3 (HL12) 5′‐AAGGGAGCT GCAGTGGAGTA‐3′, product size 200 bp (wild‐type), 500 bp (heterozygous), and 300 bp (floxed allele). All mice used in this study were in the C57BL/6J genetic background. Mice were randomly assigned to three to five mice per cage, received food and water *ad libitum,* and were housed at the UAMS AAALAC‐certified animal facility.

### Bone histology and fluorescence imaging

Freshly dissected bones were fixed in 4% paraformaldehyde overnight, washed in PBS, decalcified in 14% EDTA pH 7.1 at 4 °C for 2 weeks, and then stored in 30% sucrose solution. Bones were embedded in Cryo‐Gel (Electron Microscopy Sciences, Hatfield, PA, USA) and sectioned using CryoJane tape‐transfer system (Instrumedics Hackensack, NJ, USA) with 15 μm thickness. Frozen sections were rinsed with PBS and cover‐slipped with Vectashield mounting medium containing DAPI (Vector Laboratories Burlingame, CA, USA). Fluorescent images were acquired using Olympus BX53 fluorescence microscope (Center Valley, PA, USA) and appropriated filter set (excitation; 540/10 nm band pass filter; emission: 600/50 nm band pass filter) fluorescence microscope using a 20× lens objective.

### Isolation of bone marrow Osx1‐TdRFP^+^ cells

The tibiae and femurs were dissected from mice immediately after death. Total bone marrow cells were flushed from the bones, using a 23‐gauge needle and syringe, into ice‐cold FACS buffer containing CaCl_2_‐ and MgCl_2_‐free 1X PBS (Thermo Fisher Scientific, Carlsbad, CA, USA) and 2% FBS. Cells from individual mice in each group were centrifuged at 450 g for 6 min at 4 °C. After the red blood cells were removed with RBC lysis buffer (0.9% NH_4_Cl with 20 mm Tris base, pH 7.4), bone marrow cells were suspended in ice‐cold FACS buffer. Cells were then incubated with biotin‐conjugated rat antibodies specific for mouse CD45 (eBioscience, San Diego, CA, USA; 14‐0451, 1:100). The labeled hematopoietic cells were depleted 3 times by incubation with anti‐rat IgG Dynabeads (Invitrogen, Grand Island, NY, USA) at a bead:cell ratio of approximately 4:1. Cells binding the Dynabeads were removed with a magnetic field. The negatively isolated CD45^−^ cells were washed twice and suspended with ice‐cold FACS buffer at 1–2 × 10^6^ cells mL^−1^. Osx1‐TdRFP^+^ cells were sorted in an Aria II cell sorter (BD Bioscience, San Jose, CA, USA) using the PE‐A fluorochrome gate.

### Cell cycle analysis

CD45^−^ cells were fixed and permeabilized using fixation‐permeabilization solution (BD‐Pharmingen, San Diego, CA, USA). Subsequently, the cells were stained with anti‐Ki67‐FITC (BD‐Pharmingen #561277) and 7‐aminoactinomycin D (7‐ADD, Sigma, St. Louis, MO, USA #A9400) and analyzed by flow cytometry.

### Osteoblast differentiation

Freshly sorted Osx1‐TdRFP^−^ or Osx1‐TdRFP^+^ cells (approximately 0.1 × 10^6^/well) pooled from six mice from each group were immediately cultured with feeder layer cells (approximately 0.8 × 10^6^/well), 20% FBS, 1% PSG, and 50 μg mL^−1^ of ascorbic acid in 12‐well plates for 7 days. Half of the medium was replaced every 3 days. Cells were then cultured with 10% FBS, 1% PSG, 50 μg mL^−1^ of ascorbic acid (Sigma), and 10 mm β‐glycerophosphate (Sigma) for 21 days. For bone marrow‐derived osteoprogenitor cells, total bone marrow cells pooled from three to five mice from each group were cultured with 20% FBS, 1% PSG, and 50 μg mL^−1^ of ascorbic acid in 10‐cm culture dishes for 5 days. Half of the medium was replaced every 3 days. Mineralized matrix was stained with 40 mm alizarin red solution. To remove senescent cells selectively, bone marrow‐derived osteoprogenitor cells were collected as described above and incubated with 5 μm ABT263 (Selleckchem #S1001) in the presence of 50 μg mL^−1^ of ascorbic acid in 10‐cm culture dishes for 5 days, followed by removal of the drug. Medium was replaced every 2 days.

### Osteoclast differentiation

For co‐culture assays, red blood cell‐free bone marrow‐derived macrophages (300 000 cells cm^−2^) and stromal cells (25 000 cells cm^−2^) were seeded in 48‐well tissue culture plates with 10^−8^ m 1α,25(OH)_2_D_3_ (Sigma‐Aldrich, St. Louis, MO, USA) and 10^−7^ m PGE_2_ (Sigma‐Aldrich) in α‐MEM containing 10% FBS for 7 days. Medium was replaced every 3 days. The cells were fixed with 10% neutral buffered formalin for 15 min, and mature osteoclasts (with >3 nuclei) were enumerated after staining for TRAP using the Leukocyte Acid Phosphatase Assay kit (Sigma‐Aldrich).

### Analysis of p53 phosphorylation and γH2AX foci by immunostaining

Approximately 2000 freshly sorted Osx1‐TdRFP cells were spun on a slide for immunostaining by cytospin. After fixation with 200 μL of 4% paraformaldehyde (PFA) at room temperature for 15 min, cells were washed with cold PBS for 5 min and incubated in 200 μL of 0.5% Triton X‐100 (in PBS) at room temperature for 30 min. After washing with PBS, cells were permeabilized in 1.5% bovine serum albumin (BSA) at room temperature for 60 min and incubated overnight 4 °C with 200 μL of antibodies to p‐p53 (1:1000 in 1.5% BSA) (Cell Signaling, Beverly, MA, USA; #9284) and γ‐H2AX (1:1000 in 1.5% BSA) (Millipore , Billerica, MA, USA; #05‐636), and then incubated with the corresponding secondary antibodies in 1.5% BSA, with extensive washing between each step. The nuclear DNA of the cells was counterstained with 200 μL DAPI (0.5 μg mL^−1^ in PBS) (Sigma) at room temperature for 60 min. Cells were viewed and photographed using an Axioplan research microscope. A total of more than 100 cells per slide were counted in >30 random fields on a slide to determine the percentage of p‐p53^+^ cells and the number of γH2AX foci for calculation of the average number of γH2AX foci/cell.

### Western blot analysis

Approximately 50 000 Osx1‐TdRFP bone marrow cells or cultured bone marrow‐derived stromal cells were lysed with a buffer containing 20 mm Tris–HCL, 150 mm NaCl, 1% Triton X‐100, protease inhibitor mixture, and phosphatase inhibitor cocktail (Sigma‐Aldrich) on ice for 30 min. Protein concentration of cell lysates was determined using the DC Protein Assay Kit (Bio‐Rad, Hercules, CA, USA). The extracted protein (20–30 μg per sample) was subjected to 8–12% SDS‐PAGE gels and transferred electrophoretically onto PVDF membranes. The membranes were blocked in 5% fat‐free milk/Tris‐buffered saline for 120 min and incubate with each primary antibody followed by secondary antibodies conjugated with horseradish peroxidase. Mouse monoclonal antibodies against p21 (Santa Cruz Biotechnology, Santa Cruz, CA, USA; sc‐6246, 1:500), p‐IkB (Cell Signaling, #9246, 1:1000), β‐actin (Santa Cruz Biotechnology, sc‐81178, 1:2000), and goat polyclonal antibody for GATA4 (Santa Cruz Biotechnology, sc‐1237, 1:500) were used to detect their corresponding protein levels. Phosphorylated p65 and total p65 levels in cell lysates were determined using rabbit polyclonal antibodies for p‐p65 (Cell Signaling, #3039, 1:1000) and p65 (Abcam, Cambridge, MA, USA; ab7970, 1:1000). The membranes were subjected to Western blot analysis with ECL reagents (Millipore, Billerica, MA, USA). Quantification of the intensity of the bands in the autoradiograms was performed using a VersaDocTM imaging system (Bio‐Rad).

### Quantitative RT‐PCR (qRT‐PCR)

Total RNA for Osx1‐TdRFP bone marrow cells was extracted from freshly isolated cells using Quick‐RNA™ MicroPrep (Zymo Research, Irvine, CA, USA) according to the manufacturer's instructions. Bone marrow‐derived stromal cells were cultured in 6‐well plates, and total RNA was extracted using TRIzol reagent (Invitrogen). cDNA was obtained from 1 to 2 μg of total RNA extract using the High‐Capacity cDNA Archive Kit (Applied Biosystems, Foster City, CA, USA) according to the manufacturer's instructions. TaqMan quantitative real‐time PCR was performed using the following primers from Applied Biosystems: Runx2 (Mm00501584_m1); Col1A1 (Mm00801666_g1); Pparγ (Mm00440945_m1); p16 (Mm00494449_m1); p21 (Mm00432448_m1); Tnf‐a (Mm00443258_m1); Il‐1α (Mm99999060_ m1); Il‐6 (Mm00446190_m1); Mmp‐13 (Mm00439491_m1); and Rankl (Mm00441908_m1). Osterix and Ocn mRNA levels were determined using custom‐made TaqMan Assay‐by‐Design primer sets 5′ATCTGACTT TGCTCCCCTTAACC3′ and 5′GGGCCCTGG TTGCAAGA3′; 5′GCTGCGC TCTGTCTCTCTGA3′ and 5′TGCTTGGACATGAAGGCT TTG3′, respectively. All reactions were run in triplicate, and target gene expression was calculated by normalizing to the housekeeping gene ribosomal protein S2 (Mm00475528_m1) using the ∆Ct method (Livak & Schmittgen, 2001).

### Induction of cellular senescence by IR

To induce cellular senescence by IR, bone marrow‐derived osteoprogenitor or calvarial cells about 60–70% confluence were exposed to 10 Gy of IR in a J.L. Shepherd Model Mark I ^137^Cesium γ‐irradiator (J.L. Shepherd) at a dose of 1.080 Gy/min. Three days after irradiation cells were passaged once at a 1:3 dilution and became fully senescent 7 days after irradiation, as confirmed by SA‐β‐gal staining.

### SA‐β‐galactosidase staining

Senescent status of cells was verified by staining for SA‐β‐gal activity using a SA‐β‐gal staining kit (Cell Signaling Technology) according to the manufacturer's instructions. Senescent cells were identified as blue‐stained cells by standard light microcopy.

### Statistical analysis

For the animal experiments, no specific blinding trial was used, but mice in each group were selected randomly. The experimental sample size (*n*) of each group is described in each corresponding figure legend. Student's *t*‐test (independent samples, two‐sided) was used to detect significant aging and treatment effects, after determining that the data were normally distributed and exhibited equivalent variances. All experiments were repeated at least twice. Statistical significance was set at a *P *<* *0.05. Error bars in all figures represent SD.

## Funding

This work was supported by the National Institutes of Health [R01 AR56679 (MA), R01 CA122023 (DZ), P01 AG13918 (SCM)]; the Biomedical Laboratory Research and Development Service of the Veteran's Administration Office of Research and Development [I01 BX001405 (SCM)]; and the University of Arkansas for Medical Sciences Tobacco Funds and Translational Research Institute (1UL1RR029884).

## Author contributions

H.N.K. and M.A. designed the experiments, and H.N.K., D.Z., and M.A. analyzed the data. H.N.K., J.C., and L.S. carried out FACS analyses. H.N.K., S.I., and L.H. performed *in vitro* studies. S.C.M., C.A.O., and R.L.J. provided technical advice. H.N.K., S.C.M., C.A.O., R.L.J., D.Z., and M.A. discussed results. H.N.K., D.Z., and M.A. wrote the manuscript. All the authors revised the manuscript.

## Conflict of interest

Stavros C Manolagas is a founder and owns equity of Radius Health, Inc.
